# The unseen battle: interpreting the 2023 World Malaria Report from Burkina Faso's frontlines

**DOI:** 10.1186/s12936-024-05016-8

**Published:** 2024-06-17

**Authors:** Etienne Bilgo

**Affiliations:** 1 Institut de Recherche en Sciences de la Santé (IRSS), Direction Régionale de l’Ouest, Bobo Dioulasso, Burkina Faso; 2 Institut National de Santé Publique/Centre Muraz, Bobo Dioulasso, Burkina Faso

## Abstract

The recently released 2023 World Malaria Report sheds light on an alarming reality: despite preventive measures, malaria remains a severe issue in Burkina Faso. As researchers in the field working on malaria in Burkina Faso, the assessment suggests significant underreporting, especially in remote areas with limited healthcare access. In addition, the confusion arising from similar diseases, such as dengue, further complicates the situation. Aligning with the 2023 World Health Organization recommendations, it is time to advocate for tailored strategies in high-burden areas by emphasizing community involvement in data collection awareness campaigns for effective disease management to combat the invisible crisis lurking within communities.

**Supplementary Information:**

The online version contains supplementary material available at 10.1186/s12936-024-05016-8.

## Background

The 2023 World Malaria Report [1] is a crucial document that evaluates global progress towards malaria control and elimination. Specifically, it spotlights Burkina Faso as one of the high-burden countries, emphasizing the significant challenges it faces in combating this disease and the critical need for increased support and resources. However, the data presented in this report is believed to be underestimated for a few vital reasons.. First, many cases and deaths in remote areas, such as rural Burkina Faso, are hard to record. Second, there is the impact of auto-medication, ongoing conflicts, and crises, which can exacerbate the malaria burden. Third, there is a lack of knowledge of malaria and its prevention among the public, which prevents community-led disease control. In this paper, these realities and their impact on Burkina Faso are discussed compared to the situation reflected in the 2023 World Malaria Report*.* Personal and recent examples have been provided to highlight the critical situation of the burden of malaria in the country. Additionally, viewpoints on prospective sustainable control measures are offered. These solutions could have a powerful effect and be readily deployed and implemented to impact the disease's future management and control tools substantially.

## Main text

Globally, an estimated 2.1 billion malaria cases and 11.7 million malaria deaths were averted between 2000 and 2022. Most cases and deaths averted were in the World Health Organization (WHO) African Region (cases 82%, deaths 94%) [1]. About 96% of malaria deaths globally were in 29 countries [1]. Four countries accounted for just over half of all malaria deaths globally in 2022—Nigeria (31%), the Democratic Republic of the Congo (12%), Niger (6%) and the United Republic of Tanzania (4%) [1]. In 2022, the 12 High Burden to High Impact (HBHI) countries, which include Burkina Faso, accounted for 67% of all cases and 73% of deaths globally. Since 2018, Burkina Faso and its National Malaria Control Programme (NMCP) have followed the HBHI guidelines, which are based on three principles: (1) country-led and country-owned; (2) tailored interventions, moving away from a one-size-fits-all approach to adapt interventions to specific needs and local contexts; and (3) a swift reduction in malaria-related deaths through existing tools and prevention methods. The HBHI approach of the WHO is built on four core pillars: political commitment at all levels, strategic use of data for malaria control, enhanced technical and policy guidance, and effective coordination and leadership. It also includes two enabling environments to support these pillars. The two enabling environments are (1) strong health systems with integration of malaria control, and (2) multisectorial action for malaria. However, as indicated in the WHO 2023 report, the malaria burden has not changed much in Burkina Faso and all the other HBHI countries [1]. In Burkina Faso, since the adoption of HBHI, efforts have focused mainly on mass distribution campaigns, ensuring at least 90% of bed nets are distributed across the country, intermittent preventive treatment of malaria in pregnancy, and seasonal malaria chemoprevention (SMC) for children under five years. However, the WHO annual report still indicates around 12 million cases and approximately 5,000 deaths per year, similar to previous years. Dr. Abdoulaye Diabate, Principal Investigator of Target Malaria Burkina Faso, emphasized during the recent Grand Challenges meeting in Senegal that each statistic reflects a personal tragedy, often hidden from public view, underscoring the need for visibility [2]. Despite strategies such as insecticide-treated nets and anti-malarial campaigns, high incidence rates persist. The high resistance of mosquitoes to most of the insecticides being used exacerbates the situation. Burkina Faso's response to this crisis focuses on integrating malaria control with community health services and expanding access to preventive therapies for vulnerable groups, aiming to reduce the disease's societal impact.

As a scientist and researcher living in Burkina Faso and accessing remote areas for my fieldwork, my experiences and those colleagues often show a discrepancy in the statistics indicated in the World Malaria Reports. These discrepancies in the statistics indicated in the World Malaria Reports and those reported by the NMCP. The issue is that the case numbers measured by the national surveillance system may be inaccurate, not a flaw in the World Malaria Report. In addition, the WHO acknowledges these challenges and makes its own estimates of malaria cases in the World Malaria Report to account for them, using short modelling incidence on prevalence data from surveys. This results in a discrepancy of 8 million cases [1]. Interestingly, the modelled estimate is actually less than the reported confirmed case number, a difference worth investigating further, as it suggests there may be counterarguments to the idea that the reported case number is a dramatic underestimate. Healthcare access in these regions is minimal or non-existent, raising questions about how the report accounts for this data. Compounded by political instability, data collection efforts face significant hurdles. Malaria data from Burkina Faso's NMPC is heavily influenced by accessible areas with better healthcare access, leading to a skewed representation. This disparity underscores a critical oversight in accurately portraying the malaria situation within the country, specifically in areas mostly affected. Moreover, the lack of public knowledge about malaria and its transmission, compounded by the confusion from recent COVID-19 and dengue outbreaks, overshadows malaria cases.

Since the 2016 dengue outbreak in Burkina Faso—a disease also transmitted by mosquitoes with similar symptoms to malaria—there has been confusion even among healthcare workers about "palu-dengue," a term coined for what is perceived as a combination of malaria (paludisme in French) and dengue; however, mixed infection is rare, [3] so it is often one or the other. This has caused complications in disease identification and reporting, which was directly experienced during research conducted in remote rural areas. For example, during a recent entomological survey in rural Zorgho in Burkina Faso, a village chief who was mourning his wife who had succumbed to dengue expressed belief that the research was responsible for spreading the disease. Sadly, this false sentiment and misconception are echoed in urban areas like the capital city, Ouagadougou, and Bobo Dioulasso, where research activities sometimes face unfounded blame for spreading dengue. Even among researchers and health workers, malaria cases are underestimated or misdiagnosed, as exemplified by a recent personal experience. While attending international training in Dakar, Senegal, a feeling of being unwell arose, prompted by the symptoms and the ongoing dengue outbreak in the country recently departed from. Suspicions of contracting dengue emerged, leading to a consultation with a physician colleague who concurred and prescribed medication for dengue. However, improvement was not evident upon returning to Burkina Faso, where a diagnosis of both dengue and malaria was made, an uncommon occurrence. The levels of malaria parasites in the blood were notably high, a situation that can often result in severe disease complications (Supplementary file 1). Another example of the consequences of disease misdiagnosis was highlighted in a letter from the director of Bobo Dioulasso’s main hospital. In his letter, he requested healthcare personnel to pay attention to prescriptions because the misdiagnosis or confusion between malaria and dengue had led to several deaths among his staff and their neighbourhoods. He had also recommended the adoption of antivectorial protective methods. (Supplementary file 2) These two incidents are examples of the dangers of misdiagnosis due to the similarities of symptoms between these two diseases.

The malaria situation is worsening in Burkina Faso due to the increasing practice of self-medication by people without any scientific basis, as well as a leaning towards superstitious practices like scarification on children’s bodies. For me, the reappearance of these practices is a sign of the inadequacy of malaria control tools in delivering effective solutions to families, particularly in remote areas burdened by the relentless impact of malaria. Others have previously described the increase in perceptions and self-medication of malaria in Côte d'Ivoire when conventional prevention tools failed [4]. Beyond this, it also represents the enduring love of parents; faced with limited options to safeguard their children, they turn to superstitions as a last resort to protect them.

At the joint Laboratory of Medical Entomology and Parasitology at the Institut de Recherche en Sciences de la Santé (IRSS) and Centre Muraz in Burkina Faso, the research endeavors are fundamentally a calling to aid communities in the battle against vector-borne diseases. At the intersection of vector biology, ecology, and biotechnology, efforts are being made to develop new and complementary solutions to control mosquitoes. New solutions under development, such as Gene Drive Genetic Modified mosquitoes, will likely gain greater acceptance in operational applications [5–7]. This is because the implementation would require minimal involvement of the affected population, making them more feasible and practical. In addition, Gene Drive Modified Mosquitoes will be a more equitable method because it protects the entire community without making selections between categories of population. For instance, a notable drawback of relying on the current core protection method, bed nets, stems from its close association with population acceptance and proper usage. A similar example of such a method is the use of Genetic Modified (GM) cotton, a technique that cotton farmers widely accepted because they were experiencing the benefits of this GM-Cotton compared to conventional cotton [8, 9].

## Conclusion

Addressing the malaria crisis within our communities demands a call for enhanced community engagement and a strategic approach to data collection. A thorough analysis of Burkina Faso’s specific challenges in malaria management is crucial. Identifying adequate opportunities or strategies considering the report’s findings is essential. Leveraging the WHO operational strategy, focusing on norms and standards, innovation, strategic information, and leadership, coupled with context-based country support, is vital for a country grappling with the dual burdens of high malaria incidence and low-income status, as seen in Burkina Faso. Detailed operational plans and a results framework aim for greater transparency and accountability.

To transform the narratives surrounding malaria management, realistic statistics from high-burden areas with weaker healthcare systems, such as remote regions of Burkina Faso, must involve the collaboration of local health officials, community leaders, and citizens. Training for leaders in data recording and disease sensitization could significantly contribute to disease prevention efforts. For HBHI countries like Burkina Faso, it is believed that sustainable solutions, especially the rapid adoption of biotechnologies rooted in a profound understanding of the disease, are essential for achieving lasting change.

### Supplementary Information

Supplementary Material 1.

Supplementary Material 2.

## Data Availability

Not applicable.
