# Supplementary figures and images for: The unseen battle: interpreting the 2023 World Malaria Report from Burkina Faso's frontlines
Source: Malar J. 2024 Jun 17;23:191. doi: 10.1186/s12936-024-05016-8 (PMC11184887; doi:10.1186/s12936-024-05016-8)

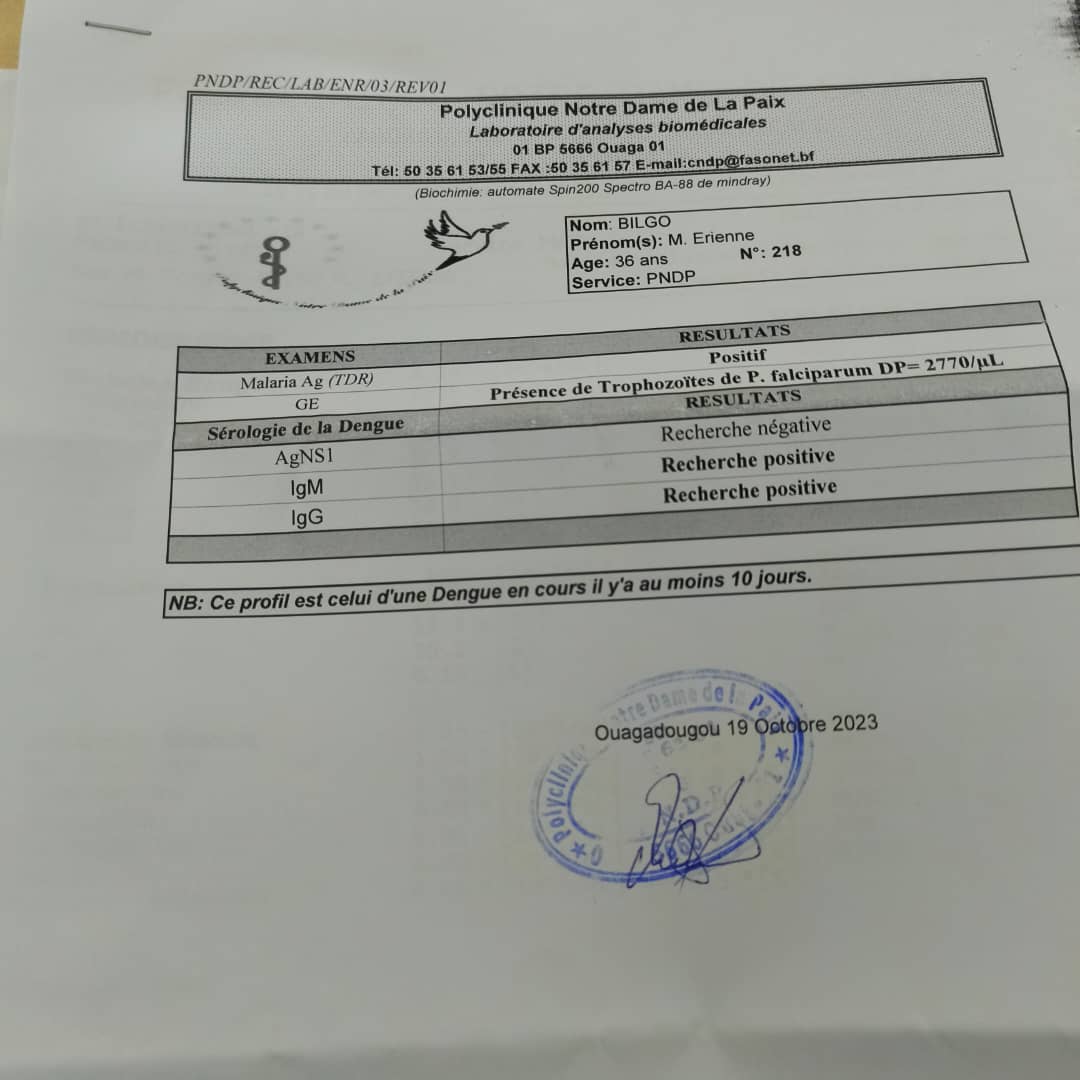

Supplement: Supplementary file 1 — Supplementary Material 1. [file 12936_2024_5016_MOESM1_ESM.jpeg]

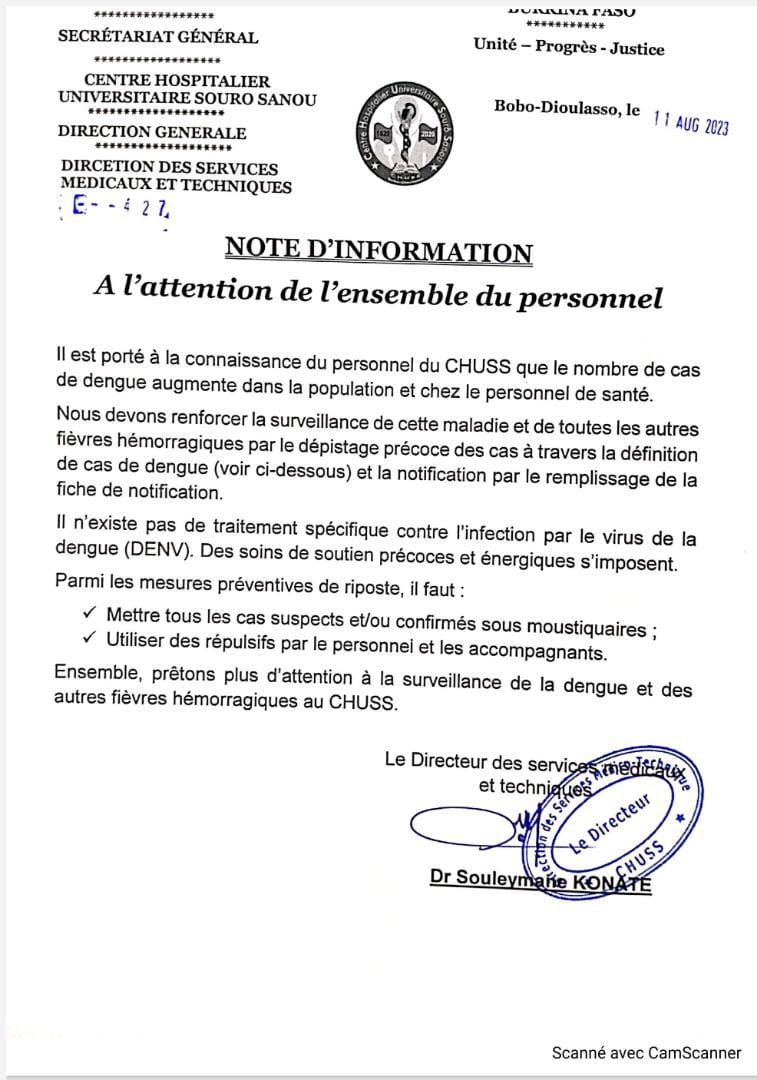

Supplement: Supplementary file 2 — Supplementary Material 2. [file 12936_2024_5016_MOESM2_ESM.jpeg]
